# Compartmental Architecture and Dynamics of Hematopoiesis

**DOI:** 10.1371/journal.pone.0000345

**Published:** 2007-04-04

**Authors:** David Dingli, Arne Traulsen, Jorge M. Pacheco

**Affiliations:** 1 Division of Hematology, Mayo Clinic College of Medicine, Rochester, Minnesota, United States of America; 2 Program for Evolutionary Dynamics, Harvard University, Cambridge, Massachusetts, United States of America; 3 Centro de Física Teórica e Computacional and Departamento de Física da Faculdade de Ciências, Lisboa, Portugal; Sanofi-Aventis, United States of America

## Abstract

**Background:**

Blood cell formation is maintained by the replication of hematopoietic stem cells (HSC) that continuously feed downstream “*compartments*” where amplification and differentiation of cells occurs, giving rise to all blood lineages. Whereas HSC replicate slowly, committed cells replicate faster as they become more differentiated.

**Methodology/Significant Finding:**

We propose a multi-compartment model of hematopoiesis, designed on the principle of cell flow conservation under stationary conditions. Cells lost from one compartment due to differentiation are replaced by cells from the upstream compartment. We assume that there is a constant relationship between cell input and output in each compartment and fix the single parameter of the model using data available for granulocyte maturation. We predict that ∼31 mitotic events separate the HSC from the mature cells observed in the circulation. Besides estimating the number of compartments, our model allows us to estimate the size of each compartment, the rate of cell replication within each compartment, the mean time a given cell type contributes to hematopoiesis, the amplification rate in each compartment, as well as the mean time separating stem-cell replication and mature blood-cell formation.

**Conclusions:**

Despite its simplicity, the model agrees with the limited *in vivo* data available and can make testable predictions. In particular, our prediction of the average lifetime of a *PIG-A* mutated clone agrees closely with the experimental results available for the *PIG-A* gene mutation in healthy adults. The present elucidation of the compartment structure and dynamics of hematopoiesis may prove insightful in further understanding a variety of hematopoietic disorders.

## Introduction

Hematopoiesis is the process for the generation of all cellular blood elements. A continuous supply of cells is necessary to compensate for the loss of cells due to apoptotic senescence or migration out of the circulating compartment. Blood cell formation has at its root hematopoietic stem cells (HSC) that have the dual property of self renewal and the ability to differentiate into all types of blood cells [1], [2], [3]. In an adult human, ∼400 HSC [4] are actively contributing to hematopoiesis, replicating at a rate of 1/year [4], [5]. On the other hand, the average daily bone marrow output in an adult human is ∼3.5×10^11^ cells [6]. Conceptually, hematopoiesis has been rationalized as a multi-step process where cell replication and differentiation are coupled with cells moving through successive stages – compartments - of maturation in a series of steps from HSC all the way down to mature blood cells. Hematopoietic cell self-renewal is not restricted to HSC but occurs in cells further downstream, albeit to a lesser extent [7]. There is ample evidence to show that as cells become more committed to a specific lineage, they replicate at faster rates up to values of more than once per day [8], [9], [10]. Thus hematopoiesis is associated with cells replicating increasingly faster as they undergo both differentiation and amplification.

In spite of such a clear-cut picture of hematopoiesis, little is known of its architectural and dynamical structure. Namely, there is no unambiguous determination of the number of stages connecting HSC and mature blood cells, let alone how fast cells replicate at each stage and to which degree amplification takes place at each stage. Such a determination has been made exceedingly difficult given the i) disconnect between morphologically distinct cell subsets and the number of divisions that a cell within a given morphologic classification can undergo [8], [9] and ii) the fact that the amplification that occurs in the bone marrow dilutes the progenitor cells making morphologic identification of these cells unfeasible. With few exceptions, many compartment (stage) specific replication rates remain undetermined despite the importance of these parameters, since the size of any compartment and its rate of replication may determine the cells’ susceptibility to malignant transformation [11]. On the other hand, previous attempts to develop mathematical models of hematopoiesis [12], [13], [14], [15], [16], [17], [18] have failed to provide such information on the number of compartments, although they suggest an architectural organization comprising from 17 to 30 stages [6], [12], [13].

Here, we develop a model that relies on a single free parameter. Despite its simplicity, the model is capable of providing i) an estimate of the number of distinct compartments, ii) the size of each compartment, iii) the specific replication rate within each compartment, iv) the mean time a given cell type contributes to hematopoiesis, v) the level of amplification which is taking place as cells differentiate and vi) the mean time separating stem-cell replication and mature blood-cell formation.

## Results and Discussion

In the following, we use Eqs. **3** and **4** derived in the Methods to explore and test the predictive capacity of our model.

During polymorphonuclear leukocyte production, ≈10^10^ myeloblasts expand and produce ≈1.4×10^11^ myelocytes in 4 steps [8], [9]. With the production of myelocytes, mitosis in the granulocyte lineage ceases and only differentiation occurs in cells further downstream (e.g. metamyelocytes) [19]. Hence, from Eq. **4** we find that the ratio of cells in compartment *i*+1 to cells in compartment *i* is 
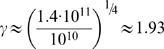
.

The parameter γ defines the net cell amplification between consecutive compartments. For γ = 1 all compartments contain the same number of cells. All larger values of γ imply an exponential growth of compartment sizes. If γ = 2, there is a net doubling of cells from compartment *i* to *i*+1.

**Figure 1:**
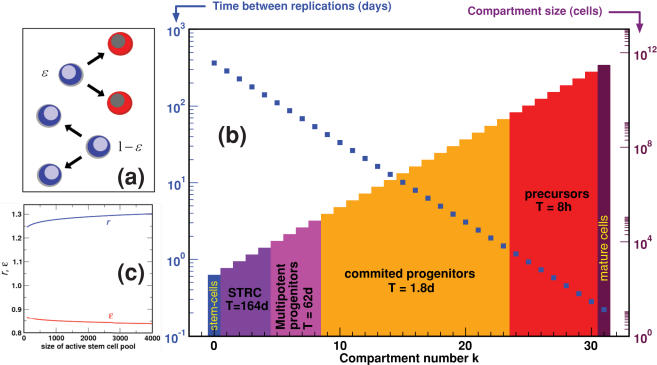
**a**) During mitosis, the parent cell may give rise to daughter cells that are different from the parent ( probability ε) being transferred downstream or, with probability 1−ε, to daughter cells identical to the parent which contribute to repopulate their own compartment. **b**) Hematopoiesis is maintained by a small group of stem cells that replicate slowly; subsequent steps lead to cell amplification and accelerated replication so that the populations grow exponentially. Contributions of each identified group of cells to hematopoiesis were computed as the weighted average based on the population distribution, being very similar to published estimates (STRC stands for short term repopulating cell, d is time in days and h is time in hours). **c**) The model parameters *r*, ε are robust to changes in the size of the active stem cell pool.

The total number of active HSC in the most primitive compartment (*N*_0_) is ∼400 [4], whereas we have ∼3.5×10^11^ as the average daily output of the hematopoietic system [6]. Here we wish to point out that our model does not dissect the branching into the different lineages and we assume that all the lineages behave in the same way. Hence, as the number of cells between two consecutive compartments increases by γ≈1.93, our model predicts that the total number of steps has to be approximately1
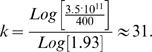
Using sequential telomere shortening as a marker of cellular replication [5], [6], it was shown that HSC within compartment *N*_0_ replicate approximately once per year. Given that granulocyte precursors can reproduce up to 5 times per day [10], for the ratio of replication rates between compartment *i* and *i*+1 we find 
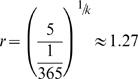
. Finally, since we know γ and *r*, we can use Eq. (4) and calculate the probability that a cell division leads to differentiation, as 
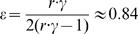
. Hence, hematopoiesis can be depicted as in Fig. 1b where from *N*_0_∼400 active HSC one reaches a per day output of *N*_31_ = 3.5×10^11^ cells after 31 stages of replication and amplification. Our results imply that most of the time, when cells in any compartment downstream of the active HSC pool replicate, they are more likely to divide symmetrically and produce 2 differentiated daughter cells than to contribute to the amplification of that compartment. In other words, our model values for ‘*r*’ and ‘ε’ are consistent with hematopoiesis being associated with an exponential expansion of cells from the HSC up to the mature cells such that, when cells are in the mitotic pool, they replicate at an accelerated rate as they differentiate. Our estimate for ε is also compatible with the observation that progenitor cells can self-renew to a limited extent [7].

Besides accounting for the multi-step nature of hematopoiesis, for which we predict a number of approximately 31 replication-amplification stages, our model also predicts the specific replication rate and size of each stage. Consequently, we may now test the predictive capacity of our model by analyzing the (unfortunately) scarce information available on hematopoiesis. To this end we evaluated data generated independently from studies on the *PIG-A* gene which codes for a subunit of the enzyme N-acetylglucosamine transferase. The *PIG-A* gene accumulates mutations at a normal somatic rate [20] and virtually every healthy adult has circulating neutrophils and erythrocytes that have lost surface expression of GPI-anchored proteins such as CD55 and CD59 [21]. The frequency of these mutant cells in healthy adults is 11–51/10^6^ neutrophils [21]. The mutations have to occur in progenitor cells such as CFU-GEMM or further downstream since they persist for variable periods of time and affect more than one lineage.

Assuming that, at any time, there is only 1 mutated progenitor, this means that adults have between 20,000 and 100,000 CFU-GEMM. According to our model, this places the CFU-GEMM, 5 to 8 steps downstream from the active HSC. Consequently, we predict that the average time these cells contribute to hematopoiesis will be between 61 and 120 days (longer for the upstream progenitors). This number is in excellent agreement with clinical observation [21]. Moreover, our model clearly predicts that individuals with the smaller clone sizes would have the clone detectable for the shorter time interval (on average), as it has to arise later in the system due to its size. This is indeed what has been experimentally observed [21]. It is noteworthy that the number of compartments, *k* = 31 is in agreement with studies where progressive telomere shortening was measured in hematopoietic progenitors and granulocytes, although these were not collected from the same individuals [6].

Finally, we assess the robustness of the results obtained. To this end, we vary the size of the active stem-cell pool repeating the determination of the parameters *r* and ε for each value. This is motivated by the fact that this is the only purely theoretical number entering Eq. **1**. Fig. 1c provides compelling evidence of the very weak dependence of the parameters *r* and ε on the actual size of the active stem-cell pool. Increasing the size up to 10000 (which, in view of the results of [4] might translate into a stem-cell pool devoid of any quiescent reserve) leads to variations of *r* and ε of only 4.4% and 2.9%, respectively.

In conclusion, we provide a simple multi-compartment model of hematopoiesis in which the observed exponential expansion of cells from the active stem cell pool to the mature cells is naturally incorporated. The model predicts the replication rate of the cells in any compartment, the size of each compartment as well as the average time cells spend in a given compartment. The model leads to numbers that fit well the limited clinical data available on humans and makes predictions which, besides being amenable to experimental validation, may provide further insight into the understanding of a variety of hematopoietic disorders. Indeed, knowledge of the size and rate of replication of cells in each compartment allows a more detailed account of the stochastic dynamics associated with myeloid disorders.

## Material and Methods

### Model

Consider a given compartment *i* with *N_i_* cells in a total of *k* compartments (stages of replication). We describe the transfer from compartment *i* to compartment *i+1* as a downstream flow of cells. When a cell in this compartment replicates, it produces two daughter cells. With a probability ε, the 2 daughter cells are exported to the downstream compartment *i*+1, leading to a decrease in the number of cells in compartment *i* to *N_i_*−1. However, with probability 1−ε, cell replication leads to identical offspring [7], [22] that remain in compartment *i*, such that *N_i_* increases by one (amplification) – see Figure 1.

Hence, these two processes change *N_i_* on average by2
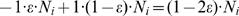
We assume that ε is the same for all compartments. In such a scenario, if cell loss from a given compartment due to export is exactly balanced by the gain from cell division (ε = 0.5), we come to the biologically implausible situation where cells from the upstream compartment are not needed at all. On the other hand, if there is no amplification (replication of cells that remain in any given compartment, ε = 1), the process works at its maximum limit of efficiency, without any reserves if a higher output of cells is temporarily required (e.g. infection or hemorrhage). In this case, the size of the compartment would be doubled in each step even for constant replication rates. Consequently, ε fulfills 0.5<ε<1.0.

In this respect, our model may appear that it only allows symmetric cell division with the two daughter cells either remaining in compartment *i* (self-renewal) or both moving to compartment *i*+1 (differentiation). However when looking at large cell populations, the average of these two processes can accommodate asymmetric cell division where the daughter cells have different fates. Indeed, for ε = 0.5, the overall population dynamics appears as originating purely from asymmetric division. In keeping with this discussion, we note that, in our current model, the most primitive hematopoietic stem cells (*i* = 0) can only divide asymmetrically (ε = 0.5) to maintain hematopoiesis without influx from any upstream compartment. A more detailed analysis that explicitly includes asymmetric cell division at the level of the individual cell has been reported elsewhere [23].

Under stationary conditions, a certain number of cells from compartment *i*−1 are required in each time step to balance the loss of cells from compartment *i* due to export. Since, per time step compartment *i* loses (2ε−1)·*N_i_*·*r_i_* cells (where *r_i_* is the rate of replication in that compartment), this number must be compensated by those cells exported from compartment *i*−1. The rate of replication in compartment *i*−1 is *r_i_*_−1_. Per time step, this compartment produces on average 2ε·*N_i_*_−1_·*r_i_*_−1_ cells. Under stationary conditions, we have (2ε−1)·*N_i_*·*r_i_* = 2ε·*N_i_*_−1_·*r_i_*_−1_, which can be written as3
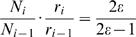



If the ratio between the replication rates of two adjacent compartments is constant (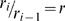), then the rates of replication grow exponentially and Eq. **2** implies that4
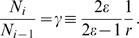
Equations **3** and **4** allow us to calculate the total number of compartments as well as ε and *r*. For 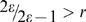 we obtain γ>1. In this biologically plausible parameter range, our model implies an exponential *growth* of the size of the compartments.
